# Decoding the circular RNA-mediated drought response in horsegram (*Macrotyloma uniflorum*)

**DOI:** 10.3389/fpls.2026.1748908

**Published:** 2026-07-29

**Authors:** Manisha Gautam, Shayista Mohiuddin, Jai Chand Rana, Amritbir Riar, Rakesh Kumar Chahota

**Affiliations:** 1 Department of Agricultural Biotechnology, Chaudhary Sarwan Kumar (CSK) Himachal Pradesh Agricultural University, Palampur, India; 2 Country Representative-India, Alliance of Bioversity International and International Center for Tropical Agriculture (CIAT), India Office, New Delhi, India; 3 Department of International Cooperation, Research Institute of Organic Agriculture (FiBL), Frick, Switzerland

**Keywords:** ABC transporters, circular RNAs, genotype-specific response, *Macrotyloma uniflorum*, stress adaptations, transcriptome analysis

## Abstract

The present study explores the drought stress responses in two genetically distinct horsegram genotypes (IC-94637 and IC-426521), with a focus on the regulation of circular RNAs (circRNAs) under controlled conditions. Experimental plants were grown in pots in a polyhouse and subjected to drought stress by withholding water. Later, physiological and molecular changes were assessed in normal and drought-stressed plants of these genotypes. Physiological parameters, including relative water content, stomatal closure, electrolyte leakage, and chlorophyll concentration, were measured. Further, a transcriptome-wide analysis was conducted to assess changes in circRNA expression under control and drought conditions. Genotype IC-94637 exhibited greater drought tolerance, as indicated by higher relative water content (79.05%), moderate stomatal closure (46%), reduced electrolyte leakage (32.57%), and increased chlorophyll levels (24.65 mg/ml). In contrast, IC-426521 showed lower chlorophyll concentration (20.01 mg/ml), increased electrolyte leakage (39.81%), reduced water content (74.53%), and higher stomatal closure (49.5%). Transcriptome analysis revealed that in IC-94637, four circRNA-related genes were expressed, of which two genes encoding ABC transporter proteins were upregulated, while the other two genes related to extensin (Ext) proteins were downregulated during drought stress. On the other hand, genotype IC-426521 exhibited expression of eight circRNA-related genes, but none of them was upregulated during drought stress. Notably, during physiological evaluations, genotype IC-94637 exhibited a rapid transition to the reproductive phase under controlled drought conditions, prioritizing seed production over extended vegetative growth, which led to early permanent wilting. This drought avoidance strategy resulted in a high seed yield despite a shortened growth period. In contrast, genotype IC-426521 showed resistance under prolonged stress but exhibited quicker wilting under acute water deficiency, indicating a distinct adaptation mechanism. Transcriptomic analysis revealed genotype-specific regulation of circular RNAs (circRNAs), suggesting differential molecular pathways contributing to drought tolerance. While differential gene expression in horsegram under drought stress has been extensively studied, this is the first report to explore the role of circular RNAs (circRNAs) under drought stress in this crop. Our findings provide deeper insights into how circRNAs contribute to stress adaptation and suggest potential functional links between circRNAs and their host genes in modulating drought tolerance in horsegram.

## Introduction

Horsegram (*Macrotyloma uniflorum*), commonly called the “poor man’s pulse,” is cultivated for both human and animal consumption across Asia and Africa (Arora and Chandel, 1972). It belongs to the genus *Macrotyloma* of the family Fabaceae (subfamily Faboideae) and is valued for its adaptability to harsh environmental conditions (Cullis and Kunert, 2017). Despite its resilience, horsegram remains an underutilized crop, often cultivated locally as part of traditional farming systems (Chahota et al., 2013; Uma et al., 2013). Cytogenetical studies revealed that it is a diploid species with a chromosome number of 2n=20 and a genome size of approximately 389 Mb (Ranasinghe and Ediriweera, 2017; Shirasawa et al., 2021).

Drought tolerance is a complex quantitative trait that is governed by multiple genes, and making it difficult to understand its genetic basis (Maazou et al., 2016). The plant response to drought stress is potentially associated with the expression of protein-coding genes, such as the elimination of reactive oxygen species (ROS) (Apel and Hirt, 2004), photosynthesis recovery (Wang et al., 2011), and stomatal adjustment (Mir et al., 2013). Recent advances in high-throughput sequencing have revealed the important role of coding and non-coding RNAs in plant stress responses. Non-coding RNAs, including long non-coding RNAs (lncRNAs) and microRNAs (miRNAs), regulate stress-responsive genes at the transcriptional and translational levels and contribute to environmental stress adaptation (Zhang et al., 2016; Khaldun et al., 2016).

Circular RNAs (circRNAs) are a class of endogenous non-coding RNAs characterized by a covalently closed structure lacking 5′ and 3′ ends. Genome-wide studies have revealed the presence and abundance of circRNAs in *Caenorhabditis elegans*, Archaea, humans, and mice, suggesting their potential roles as transcriptional regulators (Salzman et al., 2012; Danan et al., 2012; Memczak et al., 2013). In plants, however, circRNAs have been identified more recently through RNA-seq approaches. Emerging evidence indicates that circRNAs exhibit stress-specific expression patterns under various abiotic stresses. Differentially expressed circRNAs have been reported in response to dehydration stress in several plant species, including wheat, pear, maize, and *Arabidopsis* (Wang et al., 2016, 2018; Zhang et al., 2019; Zuo et al., 2018). In addition, databases such as CircNet have been developed to explore circRNA–miRNA–gene regulatory networks (Lu et al., 2015).

Despite these advances, the role of circRNAs in the drought stress response in horsegram remains largely unexplored, particularly at the genome-wide level. Considering the inherent drought tolerance of this crop, investigating circRNA-mediated regulatory mechanisms could provide valuable insights into molecular pathways associated with stress adaptation. Therefore, the present study was designed to identify and characterize drought-responsive circular RNAs in two genetically distinct horsegram genotypes (IC-94637 and IC-426521) using an *in silico* transcriptome analysis. Differentially expressed circRNAs under drought stress were identified, followed by their annotation using the horsegram genomic databases. Furthermore, the expressed circRNAs were studied for their potential regulatory roles in mediating drought stress responses. These findings contribute to the understanding of circRNA-mediated regulatory networks and provide valuable molecular resources for improving drought tolerance in horsegram breeding programs.

## Materials and methods

### Materials

**Figure 1:**
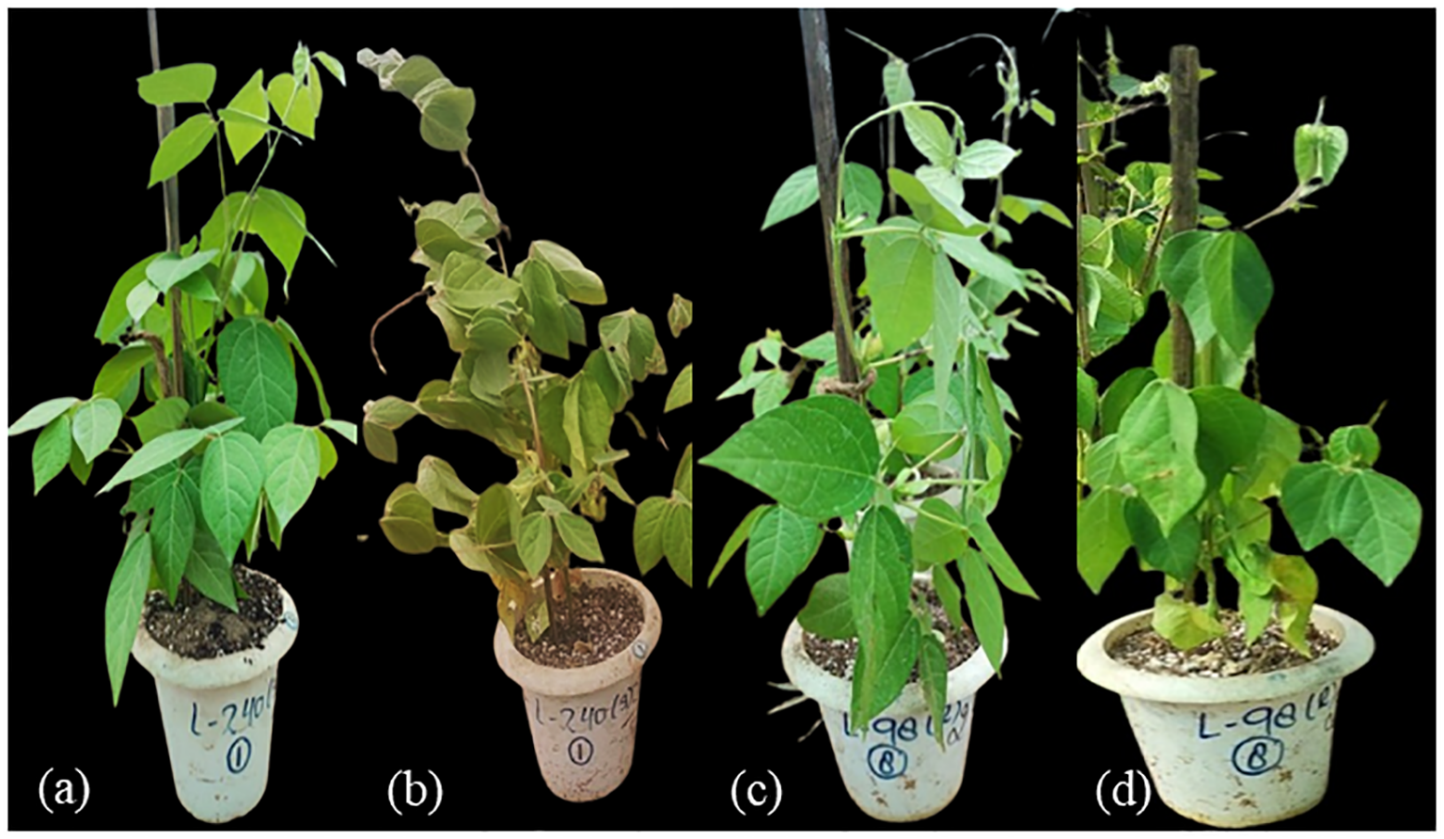
Horsegram genotypes grown in pots **(a)** IC- 426521 (L-240) - Control **(b)** IC- 426521 (L-240) -Temporary wilted **(c)** IC-94637 (L-98)–Control **(d)** IC-94637(L-98) - Temporary wilted.

Two distinct genotypes of *Macrotyloma uniflorum*, IC- 94637 (L-98) and IC-426521 (L-240), were selected for the present study (**Figure 1**). The plants were grown in the departmental polyhouse during the summer season from June to October. Seeds of both genotypes were obtained from the Department of Agricultural Biotechnology, CSK-HPKV, Palampur, Kangra, India.

### Methods

Each genotype was grown in three biological replicates in pots containing a standardized substrate mixture of sand, vermicompost, coco peat, and perlite in equal proportions (1:1:1:1) under controlled greenhouse conditions. The plants were allowed to acclimate and grow for a period of four weeks (28 days) under standard greenhouse conditions. To induce drought stress, one day prior to stress imposition the pots were fully saturated with water and then allowed to drain. After 24 h of saturation, the pots were weighed to determine the field capacity, and the soil moisture level was subsequently maintained at approximately 30% of field capacity, while water availability was estimated based on the field capacity of the soil to impose drought stress conditions. One set of pots was regularly watered, while the other set was subjected to drought stress by withholding water. Drought stress was sustained until soil moisture reached 20% of field capacity or temporary wilting was observed, corresponding to approximately 6% water availability for the plants. Shoot tissue samples were promptly flash-frozen in liquid nitrogen and stored at −80 °C until RNA extraction. RNA isolation was performed for both genotypes under normal and drought conditions using the Qiagen RNeasy Plant Mini Kit. The extracted RNA samples were subjected to sequencing conducted by NGB Diagnostics, a specialized service provider. RNA quality and quantity were assessed prior to library preparation using a NanoDrop spectrophotometer to measure concentration and purity (A260/280 and A260/230 ratios), while RNA integrity was verified by agarose gel electrophoresis. Only high-quality RNA samples (A260/280 ≈ 1.8–2.0) with intact rRNA bands were used for subsequent library preparation and sequencing.

### Measurement of physiological parameters

**Figure 2:**
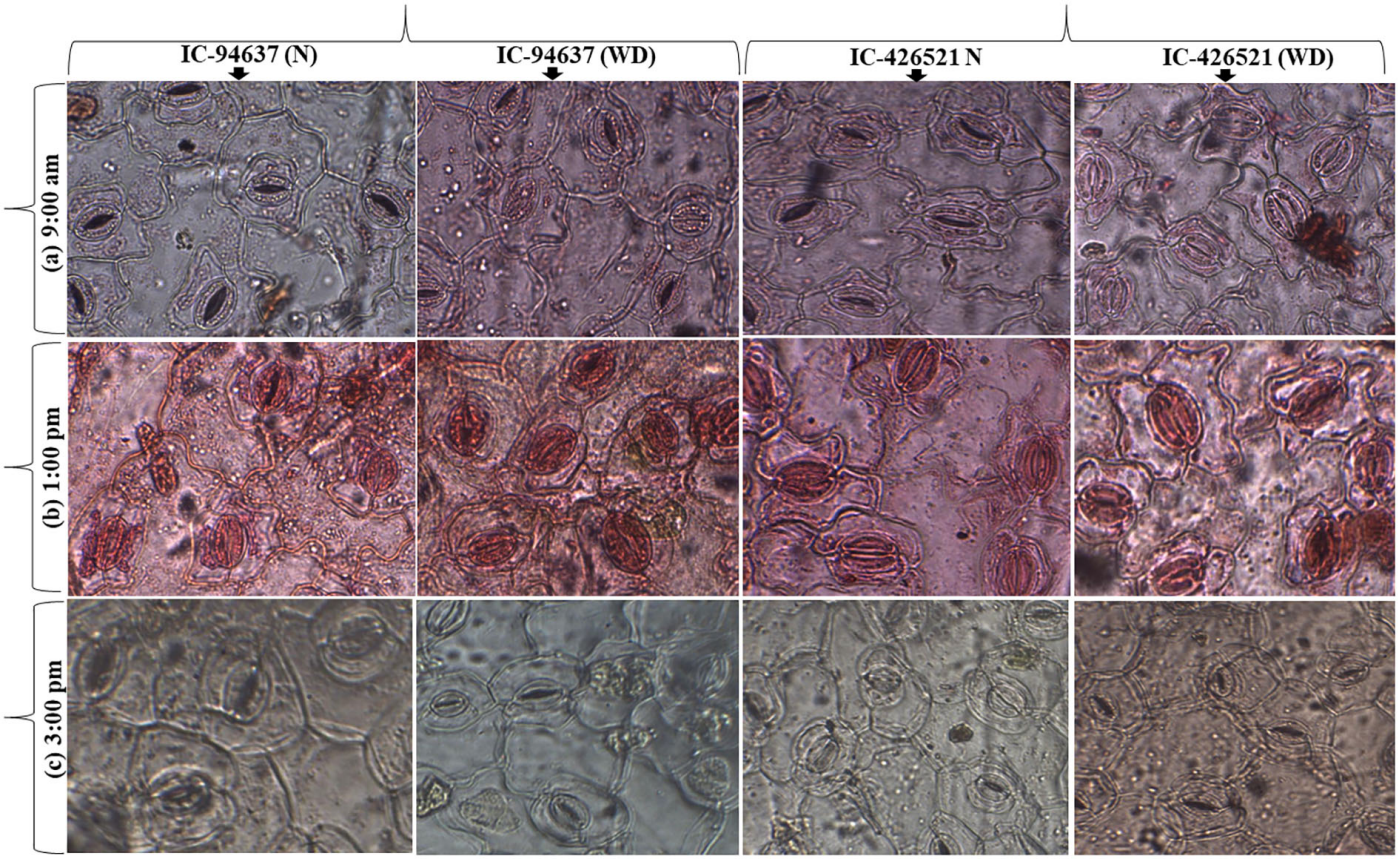
Microscopic view of stomata in the leaf tissues of horsegram genotypes (IC-426521 and IC-94637) under normal (N) and water deficit (WD conditions **(a)** at 9:00 am **(b)** at 1:00 pm **(c)** at 3:00pm.

Shoot tissue samples (three independent biological replicates, each consisting of a separate set of plants grown under identical experimental conditions) were collected daily from both normal and stressed plants for subsequent physiological assessments, including stomata count, chlorophyll index, relative water content, and electrolyte leakage percentage. For stomatal study, impressions of leaf surfaces were taken using transparent tape, and stomata were observed under a compound microscope with up to ×40 magnification (**Figure 2**). Leaf chlorophyll content was determined using 80% acetone, with absorbance recorded at 645 nm and 663 nm using a spectrophotometer (Gu et al., 2016). Relative water content (RWC) was calculated using the formula: [RWC = (fresh weight − dry weight)/(turgid weight − dry weight) × 100], with dry weight measured after incubating the turgid leaf disc for 24 h at 80 °C–85 °C. Electrolyte leakage was assessed using the formula (Ings et al., 2013): [Electrolyte Leakage (%) = [(final Conductivity)/(initial Conductivity)] × 100], and mean percentages was calculated for each sample. To monitor days to temporary wilting, visible signs were observed, and criteria were established for temporary wilting linked to soil moisture levels. Data on temporary and permanent wilting points were recorded, and statistical analysis was conducted to identify trends and variations in the time taken for plants to experience temporary wilting under controlled conditions.

### Preparation of cDNA libraries

cDNA libraries were generated through reverse transcription and subjected to high-throughput sequencing on an Illumina HiSeq platform at NCB Diagnostics Pvt. Ltd. The NEB Next Ultra II DNA Library Preparation Kit was employed for library generation, and sequencing was performed on an S4 flow cell of the Illumina HiSeq X-TEN with 159-bp paired-end chemistry. For sequencing, demultiplexing of samples and trimming of indexed adapter sequences were performed using CASAVA software. This comprehensive protocol ensured the successful extraction and sequencing of RNA from tissue samples for downstream analysis.

### Statistical analysis

The phenotypic data of the selected genotypes were subjected to statistical analysis using PAST software (version 3.25) and XLSTAT. Differences among genotypes and treatments were assessed using one-way analysis of variance (ANOVA). The analysis parameters included source of variation, sum of squares (SS), degrees of freedom (df), mean squares (MS), F-statistic, P-value, and critical F-value (Fcrit) to determine statistical significance. Significance was determined at the 5% level (*p <*0.05), and highly significant differences were determined at the 1% level (p <0.01).

### Genomic data processing

For the analysis of circular RNA expression, comprehensive bioinformatics tools were employed. Raw sequencing data were first subjected to quality assessment and read statistics generation. Then, adapter trimming was performed to obtain high-quality clean reads. Quality control of the sequencing data was also performed prior to downstream analysis to remove low-quality reads and sequencing adapters. The processed reads were aligned to the horsegram reference genome obtained from the Kazusa DNA Research Institute (KDRI) database using the Rsubread package available through CRAN. Clean reads were mapped to the reference genome to identify potential back-splicing junctions characteristic of circular RNAs. CircRNA identification was performed using a back-splice junction detection pipeline based on the alignment results. Genomic annotation files associated with the reference genome were used to classify the identified circRNAs according to their genomic origin. Based on the coordinates of the detected back-splice junctions and their overlap with annotated genomic features, circRNAs were categorized as exonic, intronic, or intergenic. CircRNAs with junction sites located within annotated exonic regions of protein-coding genes were classified as exonic circRNAs, whereas those with junctions located within annotated intronic regions were classified as intronic circRNAs. However, circRNAs with junction sites falling outside annotated gene regions were designated as intergenic circRNAs.

Differential expression analysis of circRNAs between normal and drought-stressed plants was performed using defined statistical thresholds. CircRNAs with a P value ≤0.05 and an absolute log_2_ fold change (|log_2_FC|) ≥2 were considered significantly differentially expressed. Expression patterns of the identified circRNAs were visualized using heatmaps generated with MeV. Furthermore, functional annotation of host genes associated with differentially expressed circRNAs was carried out through Gene Ontology (GO) classification and Kyoto Encyclopedia of Genes and Genomes (KEGG) pathway enrichment analysis.

## Results

### Comparative analysis of physiological parameters in response to drought stress

**Figure 3:**
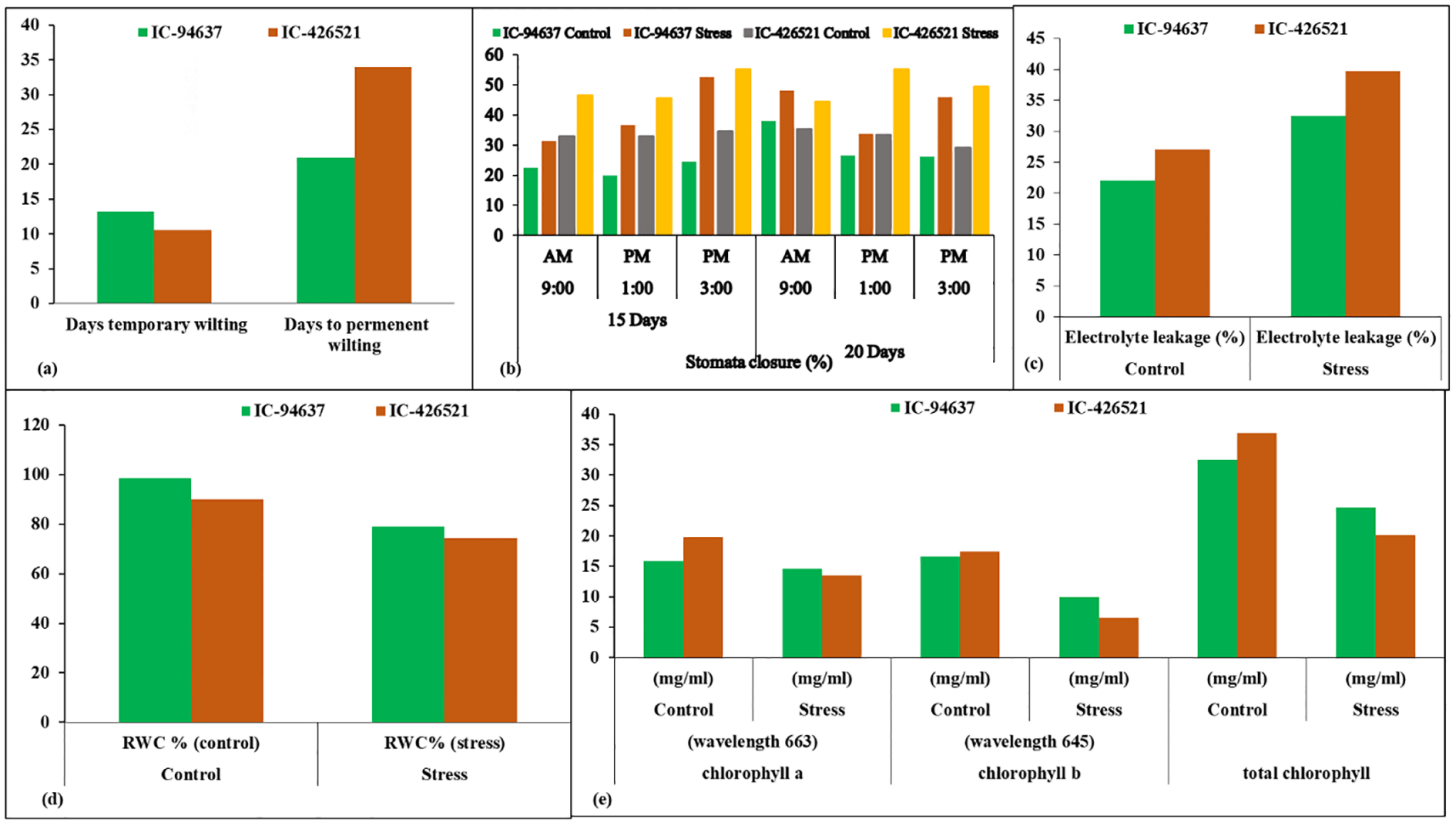
Physiological changes in IC-94637 and IC-426521 horsegram genotypes under control and drought-induced condition **(a)** Days to temporary and permanent wilting **(b)** Stomata closer % **(c)** Electrolyte leakage **(d)** Relative Water Content RWC) and **(e)** Chlorophyll content.

Comparative analysis of temporary wilting revealed that genotype IC-94637 exhibited a delay in onset of wilting, with an average of 13 days after the imposition of drought stress, whereas genotype IC-426521 showed temporary wilting at approximately 10 days (**Figure 3A**). The genotype IC-94637 exhibits a permanent wilting point after an average of 21 days of drought introduction, while IC-426521 showed permanent wilting after 34 days. This implies that IC-94637 can withstand water scarcity for a shorter duration before reaching a critical irreversible state compared to IC-426521. This difference suggests that IC-426521 may have a more rapid response to water deficiency, entering a state of temporary wilting sooner than IC-94637. Similarly, in both genotypes, stomatal closure increased significantly under drought stress compared to control conditions (**Figure 3B**). IC-426521 exhibited consistently higher stomatal closure than IC-94637 under stress, suggesting a more conservative water-saving mechanism. Specifically, in genotype IC-94637, the stomata closure increased to 46%, while in genotype IC-426521, the stomatal closure percentage increased from its initial level to 49.5% by day 20 after stress induction (**Figure 3B**). In both genotypes, closure was lowest in the morning and peaked in the afternoon (3:00 PM), reflecting diurnal variation. Additionally, stomatal closure tended to increase or remain elevated at 20 days compared to 15 days, indicating progressive stress effects over time. Analysis of variance (ANOVA) was also conducted to assess the impact of drought stress on stomatal count. Significant variations were observed at 9:00 AM (**Supplementary Table 1-1**), 1:00 PM (**Supplementary Tables 1**, **2**), and 3:00 PM (**Supplementary Tables 1**-**3**) between stressed and non-stressed plants of both genotypes. It was observed that there was a gradual increase in stomatal closure percentages for both genotypes after the introduction of stress. Initially, on day 0, there was no significant difference in stomatal closure percentages at 9:00 AM, 1:00 PM, and 3:00 PM under both conditions (**Figure 3B**). However, after 15 days of stress induction, there was a consistent increase in stomatal closure percentages during both morning and afternoon hours.

Electrolyte leakage analysis under drought stress conditions revealed that genotype IC-426521 exhibited higher electrolyte leakage (39.80%), indicating greater membrane instability and cellular damage, whereas IC-94637 showed comparatively lower electrolyte leakage (32.57%), suggesting better maintenance of membrane integrity and enhanced drought tolerance (**Figure 3C**). In contrast, IC-94637 maintained a higher relative water content (RWC) (79.05%) under stress, reflecting superior cellular hydration capacity, while IC-426521 recorded a lower RWC (74.53%), indicating reduced water retention ability (**Figure 3D**). Furthermore, drought stress imposed at the vegetative stage resulted in a significant decline in chlorophyll a, chlorophyll b, and total chlorophyll content in both genotypes (**Figure 3E**). However, IC-94637 retained a higher total chlorophyll concentration (24.65 mg/mL) compared to IC-426521 (20.01 mg/mL), suggesting a greater capacity to sustain photosynthetic pigments under stress.

### Transcriptomic analysis

**Figure 4:**
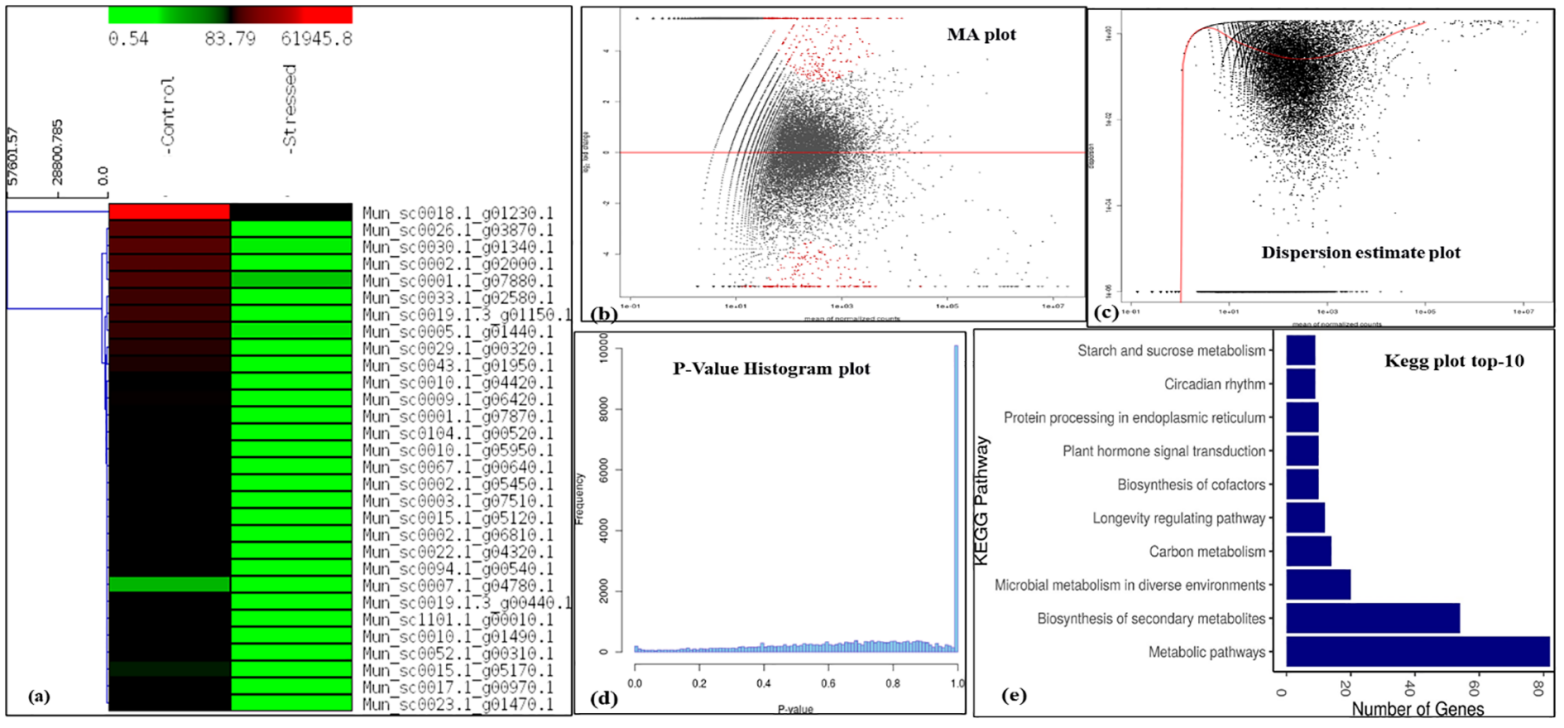
Genomic expression of IC-94637 horsegram genotype under control and drought stress: **(a)** Cluster Heat map **(b)** MA plot **(c)** Dispersion estimate plot **(d)** P-Value Histogram plot **(e)** KEGG plot.

**Figure 5:**
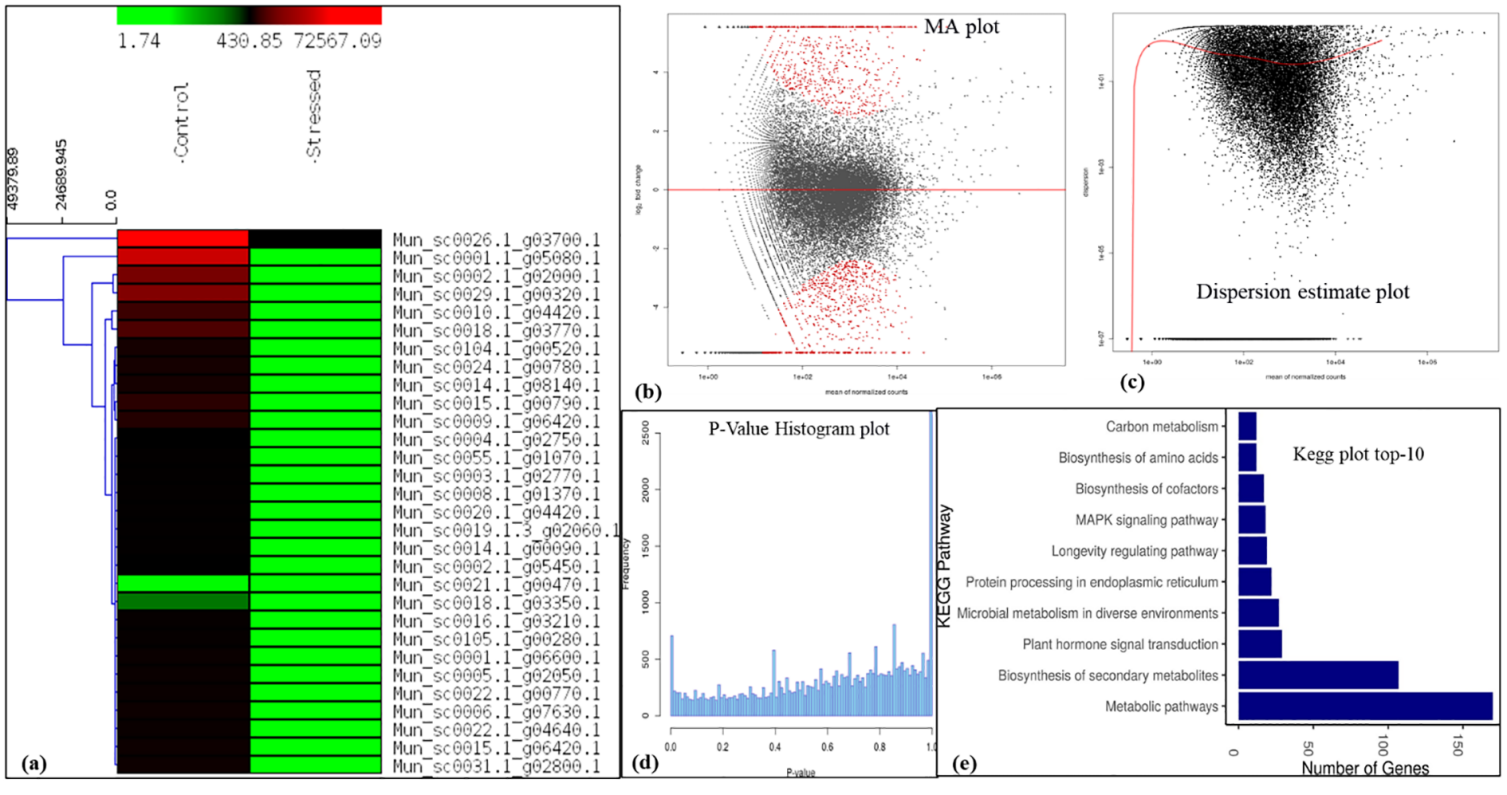
Genomic expression of IC-426521 horsegram genotype under control and drought stress: **(a)** Cluster Heat map **(b)** MA plot **(c)** Dispersion estimate plot **(d)** P-Value Histogram plot **(e)** KEGG plot.

Transcriptome sequencing generated substantial read counts for both genotypes under control and drought stress conditions. In genotype IC-94637, total reads increased from 39,909,408 under control conditions to 47,403,556 under stress. Correspondingly, the number of mapped reads increased from 15,613,072 (control) to 19,244,719 (stress), indicating successful alignment of a considerable proportion of reads to the reference sequence (**Figures 4**, **5**). In contrast, genotype IC-426521 recorded 57,777,984 total reads under control conditions, which decreased to 39,726,044 under stress. The mapped reads for IC-426521 were 25,802,758 in control and 18,383,852 under stress conditions (**Table 1**). The variations in mapped read numbers may partly reflect differences in sequencing depth rather than solely genotype-specific transcriptional responses.

### Expression and annotation profiles

The changes in transcriptomic expression in both genotypes were detected by combining expression and annotation plots. Different expression plots (cluster heatmap, MA plot, dispersion estimate plot, P-value histogram plot, and KEGG pathway analysis) were used to visualize the levels of gene expression across different samples or experimental conditions, helping to identify patterns such as upregulation, downregulation, or consistent expression. The relative analysis of circular RNA expression profiles between the two horsegram genotypes (IC-94637 and IC-426521) revealed distinct regulatory responses under drought stress. In IC-94637, the heatmap indicated a dynamic expression pattern with both upregulated and downregulated circular RNAs (**Figures 4A**, **5A**). Red and green colors indicate upregulation and downregulation, respectively, with hierarchical clustering showing distinct expression patterns, further supported by the MA plot showing a balanced distribution of significantly altered transcripts (**Figure 4B**). In contrast, IC-426521 exhibited a predominant downregulation of circular RNAs, as evidenced by the clustering pattern and the downward bias in the MA plot (**Figure 5B**). The dispersion plots in both genotypes confirmed the reliability of the differential expression analysis (**Figures 4C**, **5C**), while the P-value distributions indicated statistically significant changes (**Figures 4D**, **5D**). Functional enrichment analysis further highlighted clear differences between the genotypes, with IC-94637 showing enrichment of stress-responsive pathways, including MAPK signaling and plant hormone signal transduction, in addition to metabolic pathways. Conversely, IC-426521 primarily showed enrichment in general metabolic and biosynthetic pathways with limited activation of regulatory signaling mechanisms (**Figures 4E**, **5E**). Collectively, these findings suggest that IC-94637 possesses a more flexible and adaptive circRNA-mediated regulatory network, enabling effective drought stress responses, whereas IC-426521 displays a constrained transcriptional response, potentially reflecting lower stress tolerance.

### Differentially expressed circular RNAs

**Figure 6:**
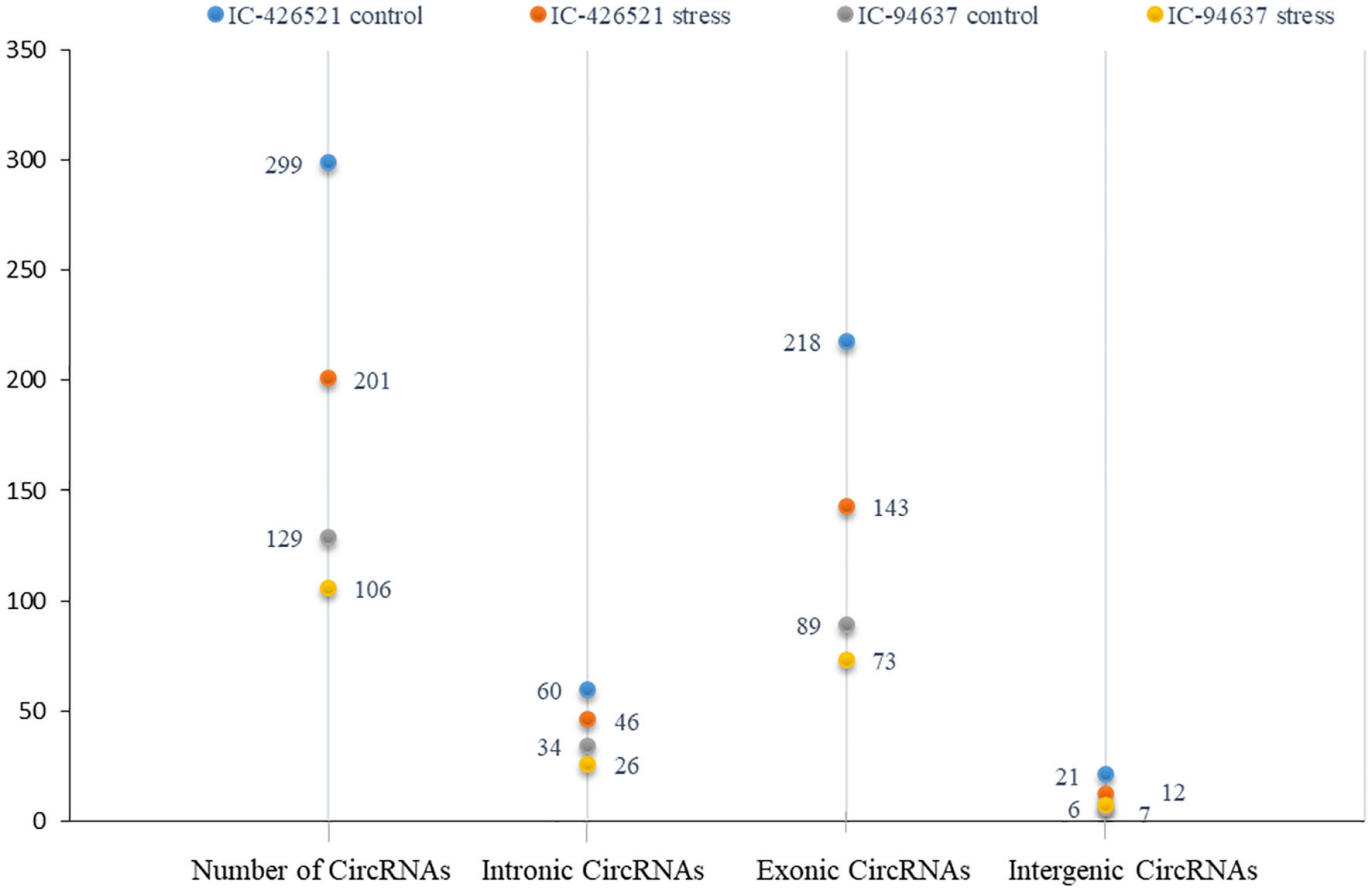
Genomic Distribution of CircRNAs in horsegram genotypes (IC-94637 and IC-426521) under control and drought-stress.

Transcriptomic analysis of horsegram was conducted to investigate variations in circular RNA levels under both drought and normal conditions. Total circular reads in IC-94637 (control) and IC-94637 (stress) numbered 440 and 796, respectively, representing sequences forming circular structures (**Table 1**). Total circular reads in IC-426521 (control) and IC-426521 (stress) were 1,316 and 691, respectively, indicating sequences forming circular structures. Line IC-426521 under normal conditions exhibited a higher number with 299 circRNAs, whereas under stress conditions the same line showed the expression of 201 circRNAs. On the other hand, line IC-94637 in control (normal conditions) displayed the lowest count with 129 circRNAs, while under stress conditions the same line exhibited 106 circRNAs (**Figure 6**). This diversity in circRNA abundance suggests potential interplay between genotype and environment for differential expression patterns. The total number of differentially expressed circular RNAs varied from 59 to 213 across different comparisons. Across all samples, a significant proportion of circRNAs were derived from intronic regions, suggesting that these circular transcripts might be products of intron retention during pre-mRNA splicing. Notably, IC-426521 (control) and IC-426521 (stress) have the highest numbers of intronic circRNAs (60 and 46, respectively), while IC-94637 (control) and IC-94637 (stress) have 34 and 26, reflecting a potential connection between intronic circRNAs and abiotic stress in these samples. These results also indicate that intronic regions may contribute significantly to circRNA biogenesis in the studied genotype. Exonic circRNAs, originating from exonic regions of protein-coding genes, also contribute substantially to the circRNA repertoire. IC-426521 (control) and IC-426521 (stress) again stands out with the highest number of exonic circRNAs (218 and 143), whereas IC-94637 (control) and IC-94637 (stress) have 89 and 73, respectively, indicating a potential association between the expression of these circular transcripts and the molecular landscape of the stress. In circular RNA expression analysis in IC-94637 and IC-426521, the total number of upregulated circular RNA and total number of downregulated circular RNA were identified. Along with these circular RNAs, many uncharacterized circular RNAs were also found. This indicates the complexity and diversity of circular RNA expression changes in different conditions. Under normal conditions in line IC-426521, eight circular RNAs were highly expressed, but under stress conditions, all circular RNAs were downregulated. In line IC-94637, three circular RNAs were expressed under normal conditions and only two were upregulated in stress conditions (**Tables 2**, **3**).

**Table 1:** Transcriptomic analysis of horsegram genotypes under control and drought-stress.

S. No.	Genotype	Total reads	Mapped reads	Circular reads
1	IC-426521 (Control)	57,777,984	25,802,758	1316
2	IC-426521 (Stress)	39,726,044	18,383,852	691
3	IC-94637 (Control)	39,909,408	15,613,072	440
4	IC-94637(Stress)	47,403,556	19,244,719	796

**Table 2:** Circular RNA regulation in IC-94637 genotype of horsegram under control and drought-stress.

Circular RNA regulation	Circ_ID	raw_IC-94637 _control	raw_IC-94637_stress	normalized_IC-94637 _control	normalized_IC-94637 _stress	logFC	logCPM	P_Value_
Top up-regulated circular RNAs	Mun_sc0013.1:4454100|4454972	9	12	120112.8796	77697.64819	-6.0114	16.47248	0.0219
	Mun_sc0310.1:14137|14748	0	8	0	32754.47839	5.827852	14.72748	0.034783
Top down-regulated circular RNAs	Mun_sc0105.1:199636|217769	11	0	60551.17229	0	-6.70261	15.12651	0.004166
	Mun_sc0105.1:217701|217991	7	0	38532.56419	0	-6.05842	14.66351	0.0219

**Table 3:** Circular RNA regulation in IC-426521 genotype of horsegram in control and drought-stress.

Circ_ID	raw_L98 _control	raw_IC-426521_stress	normalized_IC-426521_control	normalized_IC-426521_stress	logFC	logCPM	P_Value_
Mun_sc0029.1:1854990|1855253	15	0	37265.03935	0	-6.91886	14.51231	0.001409
Mun_sc0018.1:3910637|3911098	38	7	94404.76636	17390.3517	-2.41978	15.87909	0.007066
Mun_sc0105.1:218004|218315	9	0	22359.02361	0	-6.18982	13.96482	0.014079
Mun_sc0029.1:1854963|1855232	8	0	19874.68766	0	-6.02237	13.84934	0.0219
Mun_sc0002.1:8644746|8644920	7	0	17390.3517	0	-5.83289	13.72381	0.034783
Mun_sc0018.1:3907982|3908839	7	0	17390.3517	0	-5.83289	13.72381	0.034783
Mun_sc0018.1:3910133|3910456	7	0	17390.3517	0	-5.83289	13.72381	0.034783
Mun_sc0105.1:217890|218315	7	0	17390.3517	0	-5.83289	13.72381	0.034783

**Figure 7:**
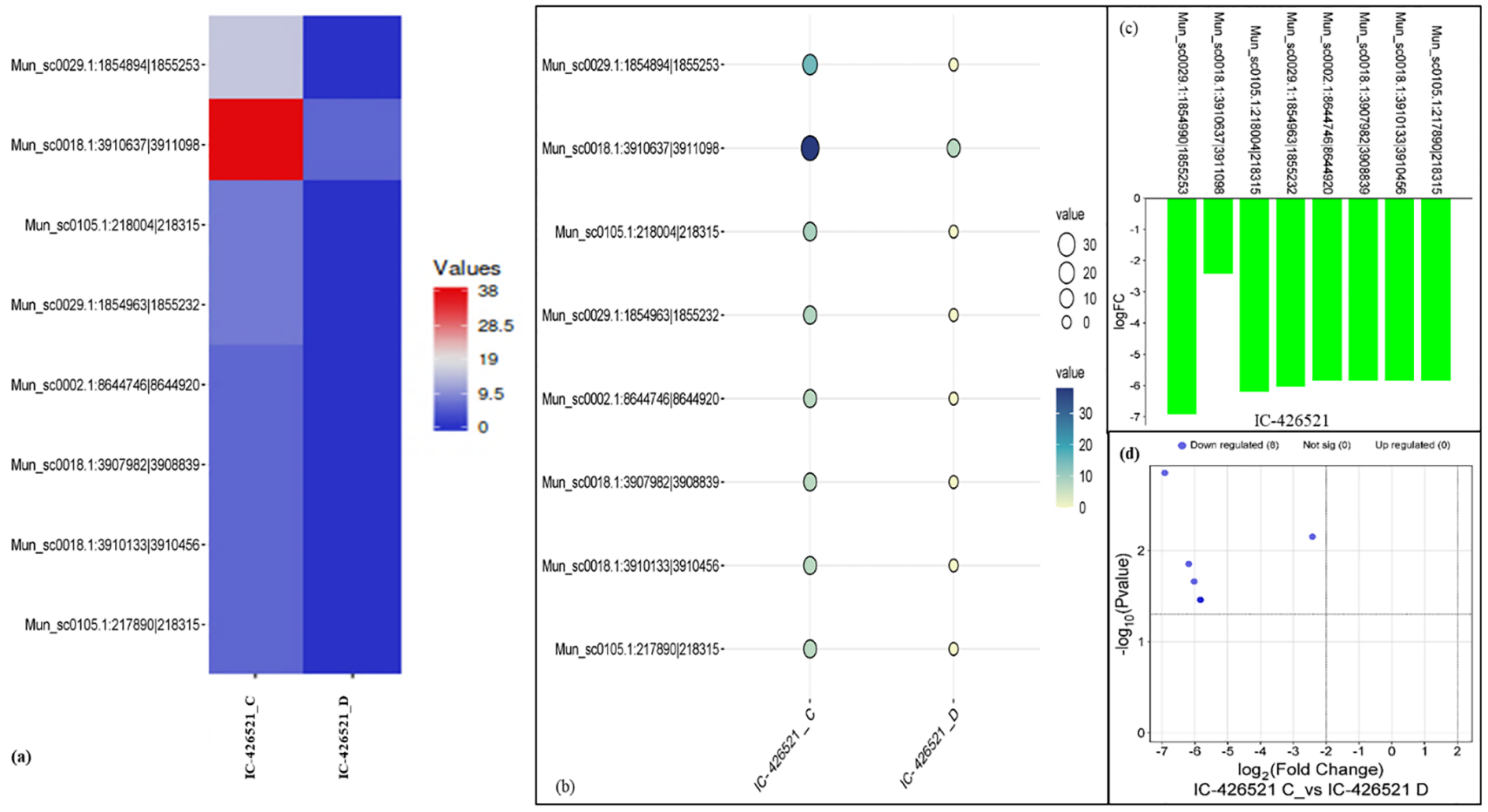
Expression of CircRNAs in IC-426521 horsegram genotype under control and drought-stress: **(a)** Heat map **(b)** Bubble plot **(c)** Gene fold change bar plot **(d)** Volcano plot.

**Figure 8:**
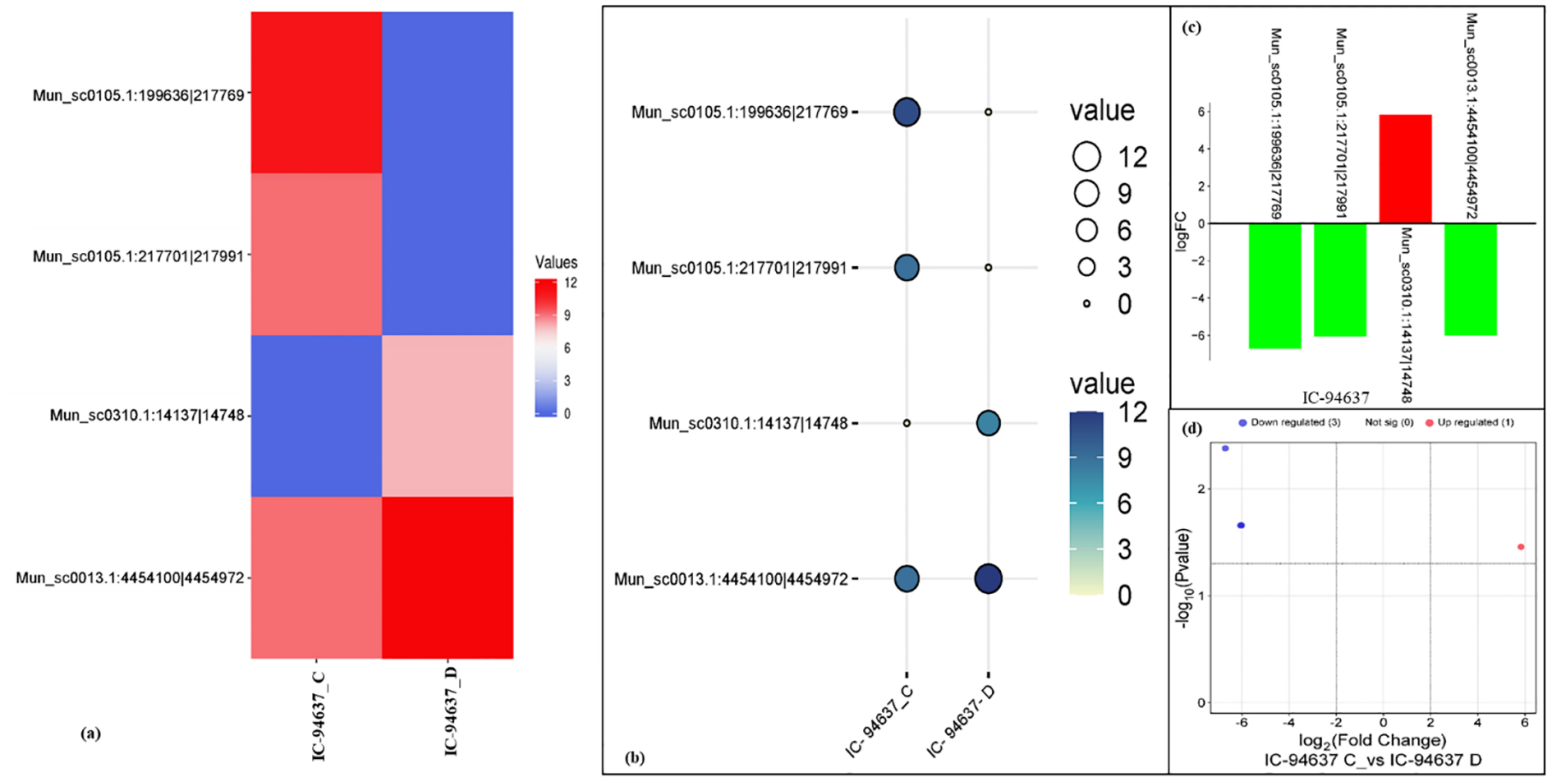
Expression of CircRNAs in IC-94637 horsegram genotype under control and drought stress: **(a)** Heat map **(b)** Bubble plot **(c)** Gene fold change bar plot **(d)** Volcano plot.

The comparative analysis of circular RNA expression between these genotypes (IC-426521 and IC-94637) under control and drought stress conditions revealed distinct regulatory patterns, which were represented by heatmaps, bubble plots, gene fold-change bar plots, and volcano plots (**Figures 7**, **8**). IC-426521 exhibited a consistent and widespread downregulation of circular RNAs (**Figures 7A, C, D** analyses), indicating a generalized suppression of transcriptional activity under stress. In contrast, IC-94637 showed a dynamic expression pattern, with both upregulated and downregulated circular RNAs, along with the presence of significantly upregulated transcripts. The heatmap analysis revealed a higher differential expression of defense-related circular RNAs in genotype IC-94637 (**Figure 8A**) compared to genotype IC-426521 (**Figure 7A**) under drought stress conditions. The bubble plots (**Figures 7B**, **8B**) further supported sustained or enhanced expression of specific circular RNAs in IC-94637 compared to IC-426521 under drought conditions. The genotype IC-94637 exhibits both positive and negative fold changes (**Figure 8C**), including at least one strongly and significantly upregulated circRNA alongside significantly downregulated transcripts, as shown in the volcano plot (**Figure 8D**). Overall, these findings suggest that IC-94637 possesses a more flexible and adaptive circRNA-mediated regulatory mechanism, whereas IC-426521 demonstrates a limited response, potentially reflecting lower drought tolerance. In the IC-94637 genotype, highly expressed circular RNAs originated from genes encoding for ABC transporter proteins, while extensin proteins (Ext genes), present in both genotypes, were found to be downregulated under drought stress conditions (**Table 4**).

**Table 4:** Significantly (adjusted pp<0.05) enriched biological process GO terms for differentially expressed genes in horsegram genotypes (IC-94637 and IC-426521).

Gene ID	Circular_ID	Gene Names	Gene Ontology (GO)	Protein names	KEGG Pathways
Mun_sc0013.1_g06650.1	Mun_sc0013.1:4454100|4454972	LOC106768245	integral component of membrane [GO:0016021]; ABC-type transporter activity [GO:0140359]; ATP binding [GO:0005524]	ABC transporter D family member 1	ABC transporters; Peroxisome;
Mun_sc0105.1_g00280.1	Mun_sc0105.1:217701|217991, Mun_sc0105.1:218004|218315, Mun_sc0105.1:217890|218315,	glysoja_046399	structural constituent of cell wall [GO:0005199]; plant-type cell wall organization [GO:0009664]	Extensin_2 domain-containing protein	–
Mun_sc0310.1_g00010.1	Mun_sc0310.1:14137|14748	–	–		ABC transporters;
Mun_sc0105.1_g00270.1	Mun_sc0105.1:199636|217769	PHAVU_004G098000g	structural constituent of cell wall [GO:0005199]; plant-type cell wall organization [GO:0009664]	Extensin-2-like	–

## Discussion

Drought stress triggers a cascade of physiological adjustments that collectively determine plant survival and productivity. Key physiological parameters such as days to temporary wilting, RWC, chlorophyll content, and electrolyte leakage were measured in the present study, revealing distinct genotypic differences in response to water deficit. The ability of plants to endure a temporary wilting phase is crucial for survival during periods of water scarcity. This phase allows the plant to conserve water and redirect resources, emphasizing its adaptive nature. The differences in the permanent wilting points of these two varieties could be attributed to variations in their genetic makeup, physiological traits, or the efficiency of their water-use mechanisms. It is important to note that the permanent wilting point represents a significant threshold, indicating the plant’s inability to recover and the onset of irreversible damage. The longer duration before permanent wilting occurs in IC-426521 suggests a higher degree of drought tolerance or resilience compared to IC-94637. However, understanding the underlying drought tolerance mechanisms solely based on wilting timelines proved challenging, as the two genotypes exhibited distinct adaptive strategies. Genotype IC-94637 responded to water stress by rapidly transitioning into the reproductive phase, bypassing extended vegetative growth and prioritizing seed production, which resulted in early permanent wilting. This drought avoidance strategy resulted in the formation of a high number of seeds, despite a shortened growth period. In contrast, IC-426521 showed a markedly different response upon encountering drought stress. It paused its reproductive development and reverted back to a vegetative state. This shift led to fewer pods and minimal seed production; yet the plants remained green and survived a significantly longer period after temporary wilting, demonstrating a drought tolerance strategy rather than avoidance.

Reduced stomatal opening under drought conditions may limit CO_2_ diffusion into the leaf and consequently reduce photosynthetic carbon assimilation, as reported in previous studies. Under severe drought conditions, stomata could entirely close, serving as an adaptive mechanism employed by tolerant genotypes to regulate carbon fixation and enhance water use efficiency (Chaves et al., 2003; Flexas and Medrano, 2002). Our findings align with those of Jiang et al. (2021), who also noted a rapid increase in stomatal closure in response to induced stress conditions. Further, the research findings of Rahbarian et al. (2011) emphasized the similar impact of water shortage on various physiological and morphological aspects of plant growth, including changes in stomatal closure. Electrolyte leakage is another parameter which is widely used to assess cell membrane integrity in plants under stress conditions. Similar results were reported by Hryvusevich et al. (2021), who demonstrated that the primary mechanisms responsible for inducing electrolyte leakage in plants involve two key processes: plasma membrane depolarization and the generation of reactive oxygen species. The precise regulation of electrolyte leakage at the level of ion channels and the selection of specific ion channel properties play a crucial role in enhancing stress resistance in higher plants (Mostofa et al., 2022). These physiological changes brought on by drought stress can be utilized to identify genotypes that are resistant or develop new crop varieties that are more productive under drought-stress conditions.

Relative water content (RWC) is also a critical physiological parameter that reflects a plant’s water status and its ability to withstand drought stress. Consistent with the present findings, Rahbarian et al. (2011) showed that water stress triggers widespread physiological and morphological changes, including a drop in relative water content (RWC), that ultimately influences plant growth and development. These morphological and physiological changes brought on by drought stress can be utilized to identify genotypes that are resistant to it or develop new crop varieties that are more productive under drought-stressed conditions. On the other hand, chlorophyll content is a key indicator of photosynthetic capacity and is often used to assess plant responses to drought stress. Similar results were reported by Mafakheri et al. (2010), who observed a reduction in chl a, chl b, and total chlorophyll under drought stress conditions in chickpea cultivars.

### Deciphering the *in silico* role of circular RNAs in plant drought stress resistance

Although there are few reports related to differential expression of various genes based on transcriptome data (Sinha et al, 2021; Bhardwaj et al, 2013), there is still no report to date on the role of circular RNA in horsegram in response to drought stress. In the present study, morphological and molecular assessment of two horsegram genotypes (IC-426521 and IC-94637) was conducted to evaluate their responses to drought stress. Circular RNAs (circRNAs) have emerged as crucial players in the regulatory networks of gene expression, with diverse functions ranging from serving as microRNA sponges (Hansen et al., 2013) to participate in protein-coding gene regulation (Zhang et al., 2013). So, in the present study, differential expression of circular RNAs (circRNAs) in these genotypes was also included to investigate the molecular mechanisms underlying their different drought response strategies. Understanding these expression patterns may provide valuable insights into the regulatory roles of circRNAs in mediating drought adaptation in horsegram. To gain deeper insights into the origin of circRNAs, a considerable proportion of circRNAs originating from intronic regions was explored, suggesting that these circular transcripts may arise during pre-mRNA splicing events, including intron retention or alternative back-splicing. These results confirm that intronic regions are likely key contributors to circRNA formation in these genotypes.

CircRNAs originating from intergenic regions, which are not associated with known genes, exhibit lower counts overall. However, their presence in all samples suggests the existence of unannotated genomic elements that may play a role in the drought-stress context. To elucidate the putative functions of circRNAs in *M. uniflorum*, we identified the parental genes by examining the genomic source positions of circRNAs. Furthermore, Gene Ontology (GO) category and Kyoto Encyclopedia of Genes and Genomes (KEGG) pathway analyses were conducted on host genes associated with differentially expressed circRNAs (DEcircRNAs) in both normal and stressed plant samples. The results revealed that these host genes are implicated in diverse biological processes, with a notable emphasis on cellular and metabolic processes. Additionally, the genes exhibited catalytic and binding activities, hinting at the involvement of circRNAs in responses to drought stress. The KEGG pathway (Kanehisa et al., 2023) enrichment analysis provided further insights, indicating a significant enrichment of host genes in pathways related to drought stress. Our findings strengthen the hypothesis that circRNAs play a crucial role in the plant’s adaptive response to environmental challenges, particularly drought. Most circular RNAs characterized in related species have been found to be responsible for plant abiotic stress adaptability (Ren et al., 2018).

The two most highly expressed circular RNAs from genotype IC-94637 were derived from genes encoding ABC transporter proteins. These proteins play a pivotal role in regulating several physiological processes under drought conditions. They modulate chlorophyll biosynthesis, facilitate the formation of iron–sulfur (Fe/S) clusters, and govern stomatal movement and ion fluxes, all of which are crucial for maintaining cellular homeostasis during water shortage. Hence, these proteins may play a central role in plant growth and developmental processes (Martinoia et al., 2002; Park et al., 2017). The enhanced expression of circRNAs originating from ABC transporter genes in IC-94637 suggests a potential upregulation of ABA transport mechanisms, which may contribute to the genotype’s improved drought resilience. These findings underscore the complex regulatory networks involving circRNAs and ABC transporters in mediating plant responses to drought stress. In *Arabidopsis thaliana*, ABCG25 and ABCG40 have been identified as ABA transporters, with ABCG25 exporting ABA from vascular tissues and ABCG40 facilitating its uptake into guard cells (Yoshiba et al., 2001). In chickpea, drought-tolerant genotypes exhibited upregulation of ABC transporters such as ABCB15-like, ABCB19, and ABCC12-like under water stress, suggesting their involvement in ABA signaling and transport, vital for drought tolerance. Additionally, DNA methylation was observed to regulate these genes, indicating an epigenetic mechanism in drought response (Yadav et al., 2025). Another category of circular RNA found in both horsegram genotype was extension (Ext) genes (Table 4). These proteins contribute to the structural integrity of the cell wall by cross-linking pectin and cellulose microfibrils, which are vital for maintaining cellular architecture during environmental stress. In the present study, the expression of genes that produce circular RNAs (circRNAs) EXT proteins was found to be downregulated in both genotypes (IC-426521 and IC-94637) under drought stress. This downregulation suggests a potential reduction in cell wall reinforcement mechanisms during water deficit conditions, which may impact the structural integrity and stress resilience of the plants. These findings highlight the complex regulatory networks involving circRNAs in mediating plant responses to drought stress. A comprehensive transcriptomic study on other horsegram genotypes (drought-sensitive; M-191 and drought-tolerant; M-249) identified conserved domains related to extensin-like regions in uncharacterized transcripts. These domains are associated with cell wall assembly and protein–protein interactions, suggesting that horsegram may utilize Ext gene expression to adapt to water stress by reinforcing its cell walls (Bhardwaj et al., 2013).

Downregulation of circular RNA (circRNA) may play a crucial role in plant responses to drought stress by influencing gene regulation, epigenetic modifications, and stress adaptation mechanisms. CircRNAs often act as miRNA sponges, preventing miRNAs from suppressing their target genes. When circRNAs are downregulated, miRNAs can function more effectively, leading to enhanced expression of drought-responsive genes. For example, in *Arabidopsis*, circ032768 was found to regulate drought tolerance by interacting with miR472, which in turn affects the expression of RPS5, a resistance gene (Yin et al., 2023). In our study, circular RNAs encoding these types of proteins were found to be downregulated in both parents, which may be responsible for the non-suppression of resistance gene associated with drought tolerance in horsegram. Studies have shown that circRNAs are associated with specific epigenetic marks that influence gene expression under drought conditions. Another role of circular RNAs was reported in maize, where drought-induced circRNAs were found linked to histone modifications, such as increased levels of H3K36me3/H3K4me1 and decreased levels of H3K9Ac/H3K27Ac (Xu et al., 2024). These modifications affect the transcriptional activity of genes involved in drought response. These findings suggest that manipulating circRNA expression could be a potential strategy for improving drought tolerance in crops in the future.

## Conclusion

The present study provides insights into the physiological and transcriptomic responses of two horsegram genotypes under drought stress. The results highlight clear differences in stress responses between the genotypes, reflected in variations in physiological parameters as well as differential gene expression patterns. Transcriptome analysis revealed several differentially expressed genes and circRNAs associated with stress-responsive pathways, suggesting their potential roles in regulating drought adaptation mechanisms in horsegram. Functional annotation further indicated the involvement of genes related to ion transport, signaling pathways, and stress-associated proteins that contribute to maintaining cellular homeostasis during water deficit conditions. In particular, gene families such as ATP-binding cassette (ABC) transporters and extension (Ext) proteins, which have been reported in other legumes to participate in stress adaptation, may also play similar roles in horsegram. Although direct functional evidence in horsegram is currently limited, the conserved nature of these genes across legume species suggests their possible contribution to drought resilience. Overall, the present study demonstrates that drought tolerance in horsegram is governed by an intricate interplay of physiological and molecular mechanisms. The contrasting strategies observed between IC-94637 and IC-426521 highlight the diversity of adaptive responses, ranging from drought avoidance to tolerance. Importantly, the integration of circRNA-mediated regulatory networks with physiological traits provides new insights into the mechanisms underlying drought resilience. These findings offer valuable targets for future functional studies and breeding programs aimed at developing climate-resilient crop varieties.

## Data Availability

The original contributions presented in the study are publicly available. This data can be found here: NCBI, PRJNA1492269.
