# Supplementary material for: Decoding the circular RNA-mediated drought response in horsegram (Macrotyloma uniflorum)
Source: Front Plant Sci. 2026 Jul 29;17:1748908. doi: 10.3389/fpls.2026.1748908 (PMC13461710; doi:10.3389/fpls.2026.1748908)
Supplement: Supplementary file 1 [file Table1.docx]

**Supplementary Information**

**Table S1** ANOVA for stomatal closure % under control and stress conditions at 9:00 AM

**Table S2** ANOVA for stomatal closure % under control and stress conditions at 1:00 PM

**Table S3** ANOVA for stomatal closure % under control and stress conditions at 3:00 PM

| **Table S1**: ANOVA for stomatal closure % under control and stress conditions at 9:00 AM | | | | | | |
| --- | --- | --- | --- | --- | --- | --- |
| **Source of Variation** | **SS** | **df** | **MS** | **F** | **P-value** | **F crit** |
| **Normal** | 13.6333 | 2 | 6.816667 | 6.591179** | 0.00266* | 3.158843 |
| **Stress** | 58.95 | 9 | 1.034211 |  |  |  |
| **Total** | 72.5833 | 11 |  |  |  |  |

*** Significance at 5% level, **significance at 1% level**

*** Significance at 5% level, **significance at 1% level**

| **Table S2**: ANOVA for stomatal closure % under control and stress conditions at 1:00 PM | | | | | | |
| --- | --- | --- | --- | --- | --- | --- |
| **Source of Variation** | **SS** | **df** | **MS** | **F** | **P-value** | **F crit** |
| **Normal** | 89.36842 | 2 | 44.68421 | 8.359956** | 0.000687** | 3.168246 |
| **Stress** | 288.6316 | 9 | 5.345029 |  |  |  |
| **Total** | 378 | 11 |  |  |  |  |

| **Table S3**: ANOVA for stomatal closure % under control and stress conditions at 3:00 PM | | | | | | |
| --- | --- | --- | --- | --- | --- | --- |
| **Source of Variation** | **SS** | **df** | **MS** | **F** | **P-value** | **F crit** |
| **Normal** | 73.73333 | 2 | 36.86667 | 10.22082** | 0.000161** | 3.158843 |
| **Stress** | 205.6 | 9 | 3.607018 |  |  |  |
| **Total** | 279.3333 | 11 |  |  |  |  |

*** Significance at 5% level, **significance at 1% level**
